# Contextual decoupling in color preference: multimodal evidence from spatial evaluation in makerspaces

**DOI:** 10.3389/fpsyg.2026.1826920

**Published:** 2026-05-15

**Authors:** Hourong Yu, Jiaqi Li, Yi Tang

**Affiliations:** 1 School of Design Art and Media, Nanjing University of Science and Technology, Nanjing, Jiangsu, China; 2 School of Big Data Engineering, Kaili University, Kaili, Guizhou, China

**Keywords:** affective-functional fit, contextual decoupling, environmental color, eye tracking, makerspace design, pupillometry, spatial evaluation, visual processing demand

## Abstract

**Introduction:**

Color preference research has mainly focused on isolated color samples, whereas architectural color is experienced within spatial and functional contexts. This study examined whether abstract hue preference remains stable when comparable chromatic conditions are evaluated in a makerspace-like interior.

**Methods:**

A three-stage design was used. Experiment 1 established baseline preferences for ten Munsell hues under D65 illumination. Experiment 2 embedded hue and saturation manipulations into a simulated makerspace and collected Preference and Comfort ratings together with eye-tracking and baseline-normalized pupillometric data under luminance control. Experiment 3 tested the stability of the findings through an online replication and a multi-viewpoint validation.

**Results:**

Preference orderings observed for isolated samples did not remain stable in the spatial context. Cooler or moderately chromatic conditions were generally rated more positively than vivid warm treatments. Saturation effects were non-monotonic, with moderate conditions outperforming the most intense treatment. Visually intense conditions attracted attention without producing more favorable evaluations, and pupillometric differences varied systematically across conditions.

**Discussion:**

These findings suggest that environmental color evaluation in cognitively demanding settings is better understood as context-sensitive spatial appraisal rather than as a direct extension of abstract hue liking.

## 1 Introduction

University makerspaces have emerged as a distinctive class of higher-education environments in which students engage in prototyping, iterative experimentation, and creative problem-solving. Unlike conventional classrooms, these spaces must support rapid shifts between ideation and focused task execution, and their physical conditions directly influence how work is approached and how long cognitive engagement can be sustained (Barrett et al., 2015; Soomro et al., 2023). In this context, the interior environment functions as a cognitive-behavioral scaffold rather than a neutral backdrop, and design variables including layout, lighting, acoustics, and color jointly shape how the space is read, used, and tolerated over time.

Among these variables, color warrants dedicated attention for three converging reasons. Chromatic atmosphere influences arousal, perceived appropriateness, and environmental comfort (Mehta and Zhu, 2009; Bower et al., 2022), which positions color as a functional rather than cosmetic parameter. Color also covers extensive architectural surfaces and is therefore encountered at a scale that swatch-based research cannot reproduce. In addition, the evidence base for color in educational interiors remains fragmented across perceptual, psychological, and design-oriented traditions (Elliot and Maier, 2014; Costa et al., 2018), which leaves designers without the same level of empirical guidance that now exists for lighting or acoustics. For makerspaces specifically, this gap is consequential, since palettes chosen to signal creativity may not be the palettes that best support sustained cognitive work (Huang et al., 2023).

A more specific problem follows from this observation and is referred to throughout as the transfer problem. A substantial body of classical color-preference research has used isolated color chips, patches, or small digital swatches (Ou et al., 2004; Palmer and Schloss, 2010; Hurlbert and Ling, 2007). That literature has mapped stable regularities in hue liking and color–emotion associations, yet treats color as a detached visual stimulus rather than as an architectural surface. In real interiors, the same hue is encountered at scale, across enclosing walls, and under expectations about the activity the room is intended to support (Higuera-Trujillo et al., 2017; Tural and Tural, 2024). Transfer from the abstract task to the spatial task is therefore neither automatic nor trivially reversible. A chip that rates highly in isolation may be excessive across a wall, and a hue that reads as unremarkable on a swatch may become acceptable in a workspace dedicated to concentration. Whether, and how far, abstract preference data can be transferred to scene-based design decisions therefore remains an empirical question that has not been resolved with multimodal evidence.

The present study addresses this transfer problem. The central research question concerns whether abstract color preference, measured under decontextualized viewing, remains a valid guide to spatial color evaluation in cognitively demanding environments, and whether subjective, attentional, and physiological indicators converge on the same answer. The investigation combines decontextualized hue preference (Experiment 1), scene-based evaluation with eye-tracking and pupillometry in a simulated makerspace (Experiment 2), and an online replication together with a multi-viewpoint validation (Experiment 3).

Five directional hypotheses were tested:

*H1*: Preference orderings obtained under abstract viewing would not remain stable when comparable chromatic conditions were evaluated as dominant spatial surfaces in a makerspace-like environment (Higuera-Trujillo et al., 2017).

*H2*: Spatial Preference and Comfort would vary nonlinearly with saturation, such that one or more moderate saturation conditions would receive more favorable evaluations than the most intense treatment (Liu et al., 2022; Weijs et al., 2023).

*H3*: Warm or visually intense conditions would attract greater gaze allocation than cooler or more moderate conditions, yet this attentional saliency would not correspond consistently to more favorable spatial evaluation (Itti and Koch, 2000; Chen et al., 2023).

*H4*: Under luminance-controlled conditions, baseline-normalized pupil dynamics would vary systematically across chromatic conditions and provide convergent physiological evidence interpretable alongside subjective and gaze-based measures (Mathôt et al., 2018; Steinhauer et al., 2022; Pan et al., 2024).

*H5*: Gender-group differences visible in abstract preference would appear less pronounced when color was evaluated in spatial context, although this effect was treated as exploratory (Hurlbert and Ling, 2007; Jue and Ha, 2022).

The expected outcome follows directly from the transfer problem. Makerspace color judgment is not expected to map directly onto abstract hue liking, and multimodal evidence spanning self-report, gaze, and pupil response is expected to converge on a more context-sensitive account. The contributions of the study are therefore twofold. Substantively, the study provides a multimodal test of color-preference transfer in a cognitively demanding educational setting, a class of interior that has not yet been examined with this combination of indicators. Methodologically, the study illustrates how abstract baselines, scene-based evaluation, eye-tracking, and baseline-normalized pupillometry can jointly discipline the interpretation of environmental color and clarify where, and how, abstract color preference diverges from spatial appraisal.

## 2 Literature review

### 2.1 Contextual decoupling reconsidered

Research on environmental design has increasingly moved beyond the view that space merely contains activity and has instead treated spatial settings as conditions that shape attention, behavior, and task engagement. This shift is especially relevant to makerspaces, where users are expected to alternate between ideation, collaboration, prototyping, and sustained problem solving rather than perform a single stable task (McCoy and Evans, 2002; Canepa et al., 2025). In such settings, interior variables are not neutral background features. They participate in how the space is interpreted, tolerated, and functionally used over time.

Within this broader context, color deserves more rigorous treatment than it has typically received in design practice. Compared with lighting, acoustics, and layout, color is often discussed in intuitive or stylistic terms, even though it occupies large architectural surfaces and contributes directly to atmosphere, perceived appropriateness, and environmental comfort (Juan and Chen, 2022; Bower et al., 2022). Innovation-oriented interiors are typically designed as differentiated environments in which material expression, enclosure, circulation, and chromatic atmosphere work together to signal different modes of use. Once makerspaces are understood in this way, color can no longer be treated as a detachable visual accent. It must be evaluated as part of a spatial and functional whole.

This broader design perspective exposes a more specific theoretical problem. The study of color preference has traditionally relied on decontextualized paradigms, in which hues are judged as isolated chips, patches, or digital swatches under controlled viewing conditions (Ou et al., 2004; Hanada, 2018; Wilms and Oberfeld, 2018). That tradition has established robust regularities in hue liking, color–emotion association, and individual variation, and frameworks such as ecological valence theory have linked abstract color preference to learned affective associations with objects and environments (Palmer and Schloss, 2010; Jonauskaite et al., 2020; Song et al., 2025). Reviews of this literature highlight both the empirical regularities and the conceptual limits of treating color as a decontextualized variable (Elliot, 2015).

A parallel line of work has shown that this distinction matters. For example, color evaluations vary systematically across sequential media, including chips, two-dimensional renderings, and three-dimensional spatial simulations, indicating that the mode of presentation is itself part of the perceptual stimulus (Ertez Ural and Yilmazer, 2010). Furthermore, context-free and in-context spatial color preferences differ not only in magnitude but also in their relationship to individual-difference variables (Akbay and Demirbaş, 2023). Evidence from rendered interiors, virtual environments, and immersive simulations points in the same direction: once color is encountered as part of an architectural scene, judgments are shaped less by abstract hue liking alone and more by atmosphere, task fit, and perceived appropriateness (Higuera-Trujillo et al., 2017; Tural and Tural, 2024; Du et al., 2023; Kalantari et al., 2021).

It is at this point that the present study adopts the term contextual decoupling. The term is not introduced as a new independent mechanism, but as an analytical label for an already observable pattern in the literature: the systematic divergence between abstract color preference, that is, color judgment without architectural embedding, and spatial evaluation, that is, judgment formed when color is encountered as part of an interior scene. Under this formulation, Preference and Comfort are treated as measured outcomes of spatial evaluation rather than as separate conceptual domains (Wilms and Oberfeld, 2018). In the same logic, visual attention is the broader construct, whereas gaze allocation refers to its eye-tracking operationalization, and saliency refers specifically to the extent to which a visual condition captures attention. This distinction matters because a salient color treatment may attract gaze without becoming more preferred or more comfortable (Rayner, 1998; Lavie, 2005). The study also uses visual processing demand as a deliberately conservative interpretive term for pupil-based differences, avoiding the stronger and often unjustified claim that pupil response is a direct readout of cognitive load (Mathôt et al., 2018; De Gee et al., 2014).

### 2.2 Multimodal evidence framework

Because contextual decoupling, as defined above, is expected to manifest at more than one level of response, the literature supporting its empirical test is correspondingly multilayered. At the evaluative level, chromatic intensity and hue can influence arousal, attentional orientation, and environmental judgment, but the direction and size of these effects depend on the setting and the task (Liu et al., 2022; Llinares et al., 2021; Xia et al., 2016). Chromatic conditions that appear stimulating in expressive or promotional settings are often experienced as excessive in environments associated with concentration, revealing that color–context fit is itself a judgment. At the behavioral level, eye-tracking research repeatedly shows that attentional capture and positive evaluation are dissociable: a visually dominant surface may accumulate long fixations simply because it is difficult to ignore, while still being judged as fatiguing or inappropriate (Chen et al., 2023; Itti and Koch, 2000). At the physiological level, pupillometry has been used to probe arousal, effort, uncertainty, and stimulus processing under luminance-controlled conditions (Laeng et al., 2012; Van der Wel and van Steenbergen, 2018; Kahneman and Beatty, 1966). Complementary physiological approaches—such as fNIRS-based measurement of stress recovery in VR-simulated interiors (Jia et al., 2025)—reinforce the value of multimodal physiological indicators for scene-based color evaluation. Current methodological guidance emphasizes that pupil dynamics are informative but not diagnostic of a single mechanism (McInnes and Sung, 2025), and recent work further shows that the relationship between arousal and pupil response is modulated by background luminance (Pan et al., 2024). These considerations frame pupillometry as a useful convergent indicator rather than a stand-alone probe of cognitive load.

**Figure 1:**
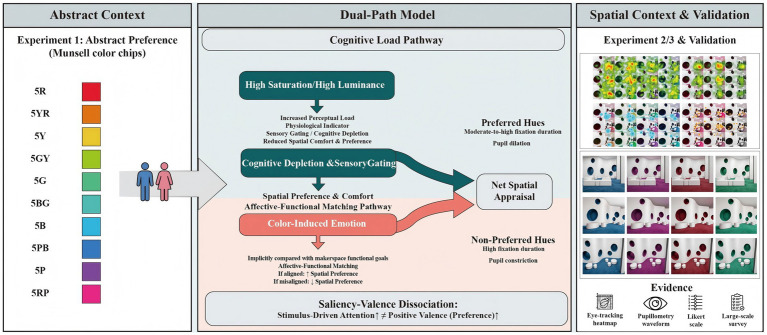
The proposed three-level dual-path model explaining contextual decoupling.

A multimodal design that integrates self-report, gaze allocation, and baseline-normalized pupil change can therefore probe contextual decoupling from three complementary angles: what participants say about a scene, how they look at it, and how their oculomotor and autonomic systems respond under matched visual conditions. Figure 1 summarizes a three-level dual-path interpretation consistent with the literature—one path concerns visual processing demand under more intense chromatic conditions, and the other concerns affective–functional appraisal of color–task fit. The figure is intended as an organizing framework for the empirical tests that follow, not as a validated mechanism, and serves as a direct bridge to the experimental design reported in Section 3.

## 3 Methods

### 3.1 Research design and participants

The study used a staged multimodal design consisting of three empirical components and one follow-up validation phase. Experiment 1 established a decontextualized baseline of hue preference using standardized color samples. Experiment 2 examined how color evaluation changed when comparable hue families and saturation conditions were embedded in a simulated makerspace interior, while subjective ratings, gaze measures, and pupil data were recorded. Experiment 3 served two validation purposes: an online replication of the spatial rating pattern and a follow-up test of whether key hue preferences remained directionally stable across alternative viewpoints.

The primary laboratory phases adopted a within-subjects design to reduce between-participant variability in esthetic judgment and physiological baseline. A total of 126 university students were recruited for Experiments 1 and 2 through campus advertisements at Nanjing University of Science and Technology. All participants were Chinese nationals enrolled full-time at Chinese universities. The final sample included 63 male and 63 female participants (M_age = 21.4 years, SD = 1.7, range 18–27). Participants were recruited from multiple disciplines, including design, mechanical engineering, business management, and the humanities, in order to reduce the likelihood that the results would reflect a narrow disciplinary esthetic profile.

**Figure 2:**
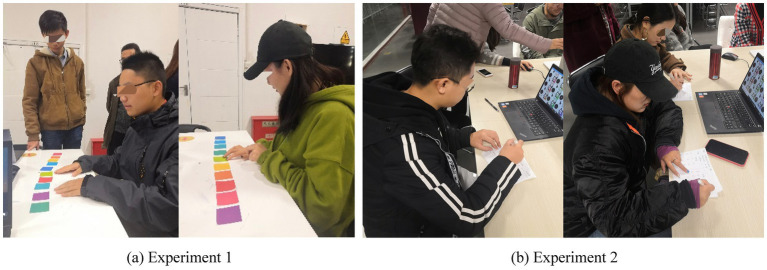
Experimental procedures of **(a)** Experiment 1 and **(b)** Experiment 2.

All participants had normal or corrected-to-normal vision. Visual acuity was verified with a standard Snellen letter chart at a 6-m test distance under even room illumination; only participants whose best-corrected acuity was 20/25 or better in each eye were retained. Color vision was screened using the 38-plate Ishihara Test for Color Deficiency (Kanehara Trading Inc., Tokyo, Japan); any participant who misidentified one or more of the diagnostic plates was excluded. An overview of the full experimental procedure for Experiments 1 and 2 is provided in Figure 2. The study protocol was approved by the relevant institutional review board, and written informed consent was obtained from all participants prior to data collection.

### 3.2 Experiment 1 establishment of abstract preference baselines

The initial phase of the study established a decontextualized baseline of color preference, deliberately restricted to hue. The logic of contextual decoupling requires that the baseline be defined on the same dimension along which the subsequent spatial comparison is drawn: because Experiment 2 evaluates hue identity at a fixed chromatic intensity (Group A), the abstract baseline was specified in the same way, so that the two experiments meet on a shared axis of variation. Saturation is reserved for the scene-based condition (Experiment 2, Group B) and is not crossed into the swatch stage. This separation is also theoretically motivated: within the present framework, chromatic intensity is not a free-standing property on which decontextualized liking can be meaningfully assessed, because its perceptual and evaluative consequences depend on the extent, geometric configuration, and functional reading of the treated surface—none of which has an architectural analogue in an isolated sample. Experiment 1 is therefore intended not to recover an innate esthetic disposition, nor to characterize hue preference as a continuous function of chroma, but to supply a controlled hue-level reference against which the later scene-based evaluations can be interpreted.

#### 3.2.1 Stimuli construction

To ensure chromatic consistency and replicability, the stimuli were derived from the Munsell color system, which is widely regarded as a psychophysical standard due to its perceptually structured hue ordering. Ten distinct hues were selected to provide full-spectrum coverage while keeping the rating task feasible within a single laboratory session. The set comprised Red 5R, Yellow-Red 5YR, Yellow 5Y, Green-Yellow 5GY, Green 5G, Blue-Green 5BG, Blue 5B, Purple-Blue 5PB, Purple 5P, and Red-Purple 5RP. This ten-hue sampling was used as a baseline palette to quantify “abstract” esthetic inclination before spatial and functional cues were introduced; the intent was to establish a stable preference ordering rather than to estimate a continuous hue function. Detailed parameters, including Munsell notations and the corresponding sRGB values converted for digital consistency, are provided in Table 1. Physically, these hues were produced as matte-finish color cards measuring 6 centimeters by 6 centimeters. The matte finish was selected to minimize specular reflections and highlight hue-based judgments under controlled illumination.

**Table 1:** Nearest-family mapping between Experiment 1 Munsell hues and Experiment 2 HSL-rotated spatial stimuli (A1–A12).

No.	Munsell hue notation	English name	Approximate sRGB value	Color patch example	HSL value	Exp2 condition
1	5R	Red	(230, 50, 60)	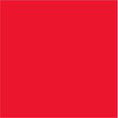	(357, 78, 55)	A1/A5
2	5YR	Yellow-Red	(245, 130, 50)	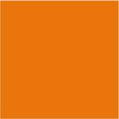	(25, 91, 58)	A9
3	5Y	Yellow	(245, 215, 70)	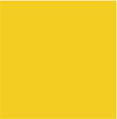	(50, 90, 62)	A2
4	5GY	Green-Yellow	(195, 220, 70)	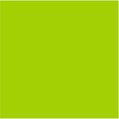	(70, 68, 57)	A6
5	5G	Green	(70, 175, 115)	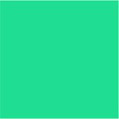	(146, 43, 48)	A10
6	5BG	Blue-Green	(60, 185, 175)	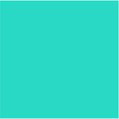	(176, 51, 48)	A3
7	5B	Blue	(55, 125, 195)	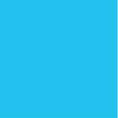	(210, 56, 49)	A12
8	5PB	Purple-Blue	(90, 90, 180)	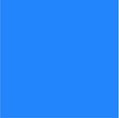	(240, 38, 53)	A8
9	5P	Purple	(155, 70, 165)	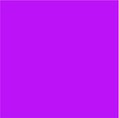	(294, 40, 46)	A4/A11
10	5RP	Red-Purple	(210, 75, 135)	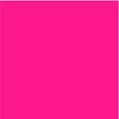	(333, 60, 56)	A7

#### 3.2.2 Procedure

The evaluation took place in a dedicated color laboratory booth equipped with D65 standard daylight illuminants to ensure color rendering accuracy. Participants were seated at a comfortable distance and each color card was presented on a neutral gray background. Participants were instructed to evaluate each hue as an abstract entity divorced from any specific object or functional application, relying only on their immediate intuitive reaction. Preference was rated on a single-item 5-point Likert scale adapted from the color preference literature (Hanada, 2018; Palmer and Schloss, 2010); the five ordinal anchors were 1 = strongly dislike, 2 = dislike, 3 = neutral, 4 = like, 5 = strongly like. A single-item format was used for two reasons. First, Experiment 1 was designed to provide a directly comparable decontextualized baseline to the analogous single-item Preference rating used in Experiment 2, so a matched response format reduced cross-experiment measurement noise. Second, abstract color liking is treated in the literature as a one-dimensional evaluative judgment rather than a multidimensional construct (Palmer and Schloss, 2010), which makes a composite multi-item scale unnecessary at this stage. The anchor labels and numerical values were printed on a card placed next to each color sample; participants indicated their response verbally, which was recorded by the experimenter. Two achromatic practice trials preceded the main task to familiarize participants with the scale. The resulting ratings constituted the scalar dataset used for subsequent statistical comparison with spatial preference outcomes.

### 3.3 Experiment 2: spatial simulation, eye tracking, and pupillometry

#### 3.3.1 Stimulus construction

Because Experiment 1 used physical Munsell chips evaluated under D65 illumination, and Experiment 2 used digital HSL-based wall renderings, a principled procedure was used to map between the two systems. For each Munsell hue, the chip was first characterized by its published CIE xyY values under the C/2° illuminant and chromatically adapted to D65 using the Bradford transform. The D65 XYZ values were then converted to sRGB, gamut-clipped where necessary, and finally converted to the HSL coordinates reported in Table 1. Because the two color systems are not metrically equivalent, Table 1 is used as an interpretive family-level correspondence between experiments rather than as a basis for pointwise colorimetric comparison. Cross-experiment inferences are accordingly restricted to broad hue-family correspondence throughout Sections 4 and 5.

**Figure 3:**
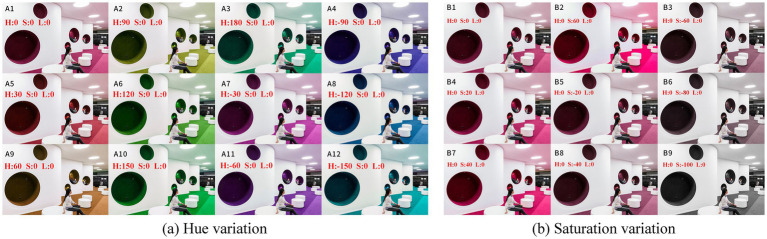
**(a)** Hue variation and **(b)** saturation variation stimuli for Experiment 2.

Experiment 2 examined how color evaluation changed when the same broad hue families were embedded in a makerspace-like interior scene rather than presented as isolated samples. The stimuli were derived from a high-resolution image of the Aalto University Learning Centre, selected as a visually plausible reference scene because it contained open circulation, collaborative zones, and large wall surfaces suitable for controlled color manipulation. The image served as a standardized spatial template rather than as an empirical representation of a single “ideal” makerspace. Color manipulations were implemented in Adobe Photoshop using the HSL color model, and all modified stimuli were generated from the same base image to preserve spatial composition across conditions. Figure 3 presents the two stimulus sets used in this experiment.

Group A was designed to isolate hue variation. The wall surfaces were rotated in 30° increments around the HSL hue circle while saturation and lightness were held constant, generating 12 spatial color conditions that spanned the full hue range. This manipulation provided a symmetric and digitally controllable hue set for scene-based evaluation. Because Experiment 1 was based on Munsell chips whereas Experiment 2 used HSL-based scene rendering, the linkage between the two experiments was interpreted only at the level of broad hue families rather than as a one-to-one colorimetric equivalence. Table 1 was therefore used only as an interpretive correspondence table and not as the basis for direct parametric comparison across color systems.

Group B was designed to examine the effect of chromatic intensity under a fixed hue. A single wall-color hue was retained, and saturation was adjusted in 20% steps from −100 to +60%, generating nine saturation conditions. The −100% condition produced an achromatic grayscale version of the manipulated wall region, whereas the +60% condition represented the most intense chromatic treatment retained for formal testing. Higher saturation levels were not used because pilot rendering produced visible artifacts and reduced spatial realism. This manipulation allowed saturation to vary while holding scene geometry, hue identity, and lightness constant. Figure 3b illustrates the resulting graded saturation series.

#### 3.3.2 Apparatus and recorded measures

Eye-movement and pupil data were recorded using a Tobii X60 remote eye tracker operating at 60 Hz and mounted below a 24-inch calibrated monitor (1,920 × 1,080 resolution). Participants were seated approximately 60 cm from the display. The sampling rate was sufficient for the fixation-based and trial-level pupil measures used in the present study, although it was not intended for fine-grained microsaccadic analysis.

Two categories of dependent measures were derived. The first category comprised subjective ratings of Preference and Comfort, both recorded on 5-point Likert scales immediately after each trial. The second category comprised oculomotor and pupil measures. For gaze behavior, fixation count and total fixation duration were extracted from predefined areas of interest located on the manipulated wall surfaces. For pupillometry, the primary outcome was baseline-normalized pupil change summarized at the trial level. In line with current methodological recommendations (Steinhauer et al., 2022), pupil variation was interpreted conservatively as reflecting a composite response associated with visual intensity and task-related processing, rather than as a direct one-to-one measure of cognitive load.

Heatmaps and scanpath maps were generated as descriptive visualizations to illustrate gaze allocation patterns across conditions. These visual outputs were used to support interpretation of the rating and AOI-based findings but were not treated as stand-alone inferential statistics. This distinction was important because visually salient regions may attract attention without necessarily improving spatial evaluation.

#### 3.3.3 Luminance control and preprocessing

Because pupil diameter is highly sensitive to luminance variation, luminance control was applied to the manipulated wall region before data collection. A fixed wall mask was defined on the reference scene and applied identically to all stimuli so that the manipulated region remained geometrically constant across hue and saturation conditions. Original sRGB images were converted to linear RGB and then transformed to CIE XYZ space, with the Y channel used as the luminance metric (Epicoco et al., 2024). Pixel values within the masked wall region were adjusted so that the manipulated area remained closely matched across conditions in mean luminance while preserving the intended hue or saturation differences. The acceptance criterion for the rendered set was an absolute mean-Y deviation below 2% relative to the reference image, with no visible banding after conversion back to sRGB. Room illumination, monitor brightness, and viewing distance were held constant throughout testing.

Pupil data were segmented by trial and baseline-normalized using the final 2 s of the neutral gray fixation screen preceding stimulus onset. Blink segments were identified using Tobii validity flags. Trials with more than 30% missing pupil samples were excluded from pupil analysis. For the remaining trials, short blink-related gaps were linearly interpolated, and a low-pass smoothing procedure was applied to reduce high-frequency noise, following standard preprocessing practice (Mathôt et al., 2018; Brisson et al., 2013). To minimize contamination from the initial pupillary light reflex, the primary pupil metric was the mean baseline-normalized pupil change calculated over the 1–8 s viewing interval. This trial-level summary measure was selected to support condition-wise comparison rather than fine-scale time-course modeling.

#### 3.3.4 AOI definition and experimental procedure

Areas of interest (AOIs) were defined on the wall surfaces whose colors were experimentally manipulated. The AOI mask was created once on the reference image and then applied unchanged to all hue and saturation stimuli so that gaze measures were based on the same spatial region across conditions. Elements not directly related to the wall-color manipulation, including windows, furniture, signage, occupants, and strong highlights, were excluded from the AOIs. As a result, fixation count and total fixation duration reflected attention directed specifically to the color-treated architectural surfaces rather than to other visually salient objects in the scene.

After a standard 9-point calibration, participants completed the task individually in a dim and quiet laboratory setting. They were instructed to imagine entering the displayed makerspace to carry out a semester-long innovation project and to evaluate the environment in terms of how suitable and comfortable it would be for sustained work. Each trial began with a 2 s neutral gray fixation screen, followed by an 8 s presentation of one stimulus image. Immediately after image presentation, participants rated the scene on two 5-point scales: Preference and Comfort. The order of the 21 stimuli was randomized for each participant. The 8 s viewing period was selected on the basis of pilot testing so that participants had sufficient time to visually inspect the scene without introducing excessive fatigue.

### 3.4 Experiment 3: online replication and viewpoint-robustness validation

#### 3.4.1 Online replication of the spatial preference pattern

Experiment 3 was conducted to examine whether the spatial preference pattern observed in the laboratory could be reproduced in a broader and less tightly controlled viewing context. The same image set used in Experiment 2 was distributed through an online survey platform to an independent sample of 112 university students recruited from multiple institutions, including Nanjing University of Science and Technology and Tianjin University. Participants rated the stimuli using the same Preference and Comfort scales as those used in the laboratory study. No eye-tracking or pupil data were collected in this phase. Because display conditions could not be standardized online, this experiment was not treated as a substitute for the laboratory data; instead, it served as an external replication of the directional pattern observed in the scene-based rating task.

#### 3.4.2 Follow-up validation across spatial viewpoints

**Figure 4:**
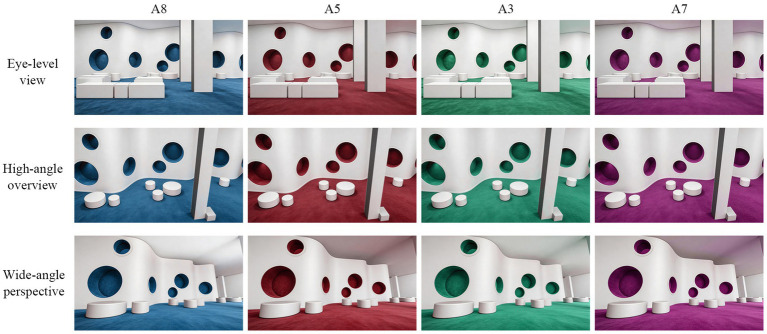
Validation stimuli displaying four target hues across three distinct spatial perspectives.

A further validation study was conducted to test whether the main hue preference pattern depended unduly on the fixed viewpoint of the original photographic scene. For this purpose, a generic makerspace model was rendered in 3D software and four hue conditions were selected for robustness testing. Two of these conditions, A8 and A5, represented the more positively evaluated spatial colors identified in the earlier experiments, whereas A3 and A7 served as lower-rated comparison conditions. Each of the four target hues was rendered from three viewpoints: an eye-level perspective, a high-angle overview, and a wide-angle view, producing 12 validation stimuli in total. These stimuli are shown in Figure 4.

An independent sample of 68 participants evaluated the 12 viewpoint-varied renderings using the same rating criteria. The purpose of this follow-up phase was not to reproduce the full multimodal protocol of Experiment 2, but to determine whether the broad spatial preference ordering remained visible when the same color conditions were viewed from different spatial perspectives. This design allowed the study to distinguish color-related evaluation from a possible artifact of a single camera composition.

### 3.5 Data analysis

All statistical analyses were performed in IBM SPSS Statistics 27 and in R 4.3.1 using the lme4 and lmerTest packages; effect sizes and bootstrap confidence intervals were computed with the effect size package. Before inferential testing, the assumptions of the repeated-measures framework were examined. Normality of residuals was checked with Shapiro–Wilk tests and visual inspection of Q–Q plots at the participant and condition level; where mild departures from normality were observed, we relied on the robustness of mixed-effects models to non-normal residuals given the large trial count. Sphericity for the repeated-measures ANOVA component was assessed using Mauchly’s test; when it was violated (*p* < 0.05), the Greenhouse–Geisser correction was applied to the degrees of freedom. Homogeneity of variance across conditions was examined using Levene’s test.

For Experiment 2, Preference and Comfort were analyzed using linear mixed-effects models with Condition as a fixed effect and a random intercept for Participant. More complex random-effects structures were considered only when they improved model fit (ΔAIC > 2) without producing singular fits or convergence problems. Omnibus F-tests were obtained via Satterthwaite approximations to degrees of freedom and are reported in Table 2 alongside partial *η*^2^ with bootstrap 95% confidence intervals. AOI-based fixation count, total fixation duration, and trial-level baseline-normalized pupil change were analyzed using the same mixed-effects structure.

**Table 2:** Omnibus fixed-effect tests for condition effects in repeated-measures analyses.

Outcome	Fixed effect test	df	Test statistic (F)	*p*	Effect size (*η*_p_^2^)	95% CI
Preference	Hue	11, 1,500	55.62	<0.001	0.29	[0.25, 0.33]
Comfort	Hue	11, 1,500	48.31	<0.001	0.26	[0.22, 0.30]
Fixation count	Hue	11, 114	452.18	<0.001	0.81	[0.75, 0.86]
Fixation duration	Hue	11, 114	473.51	<0.001	0.82	[0.76, 0.87]
Pupil change	Hue	11, 114	85.07	<0.001	0.45	[0.38, 0.52]
Preference	Saturation	8, 1,125	155.20	<0.001	0.52	[0.48, 0.56]
Comfort	Saturation	8, 1,125	142.65	<0.001	0.50	[0.45, 0.55]
Fixation count	Saturation	8, 117	421.32	<0.001	0.85	[0.81, 0.89]
Fixation duration	Saturation	8, 117	456.37	<0.001	0.88	[0.84, 0.92]
Pupil change	Saturation	8, 117	3325.69	<0.001	0.96	[0.94, 0.98]

For the hue manipulation, the analytical objective was to determine whether the spatial rating pattern diverged from the abstract preference ordering established in Experiment 1. Because the Munsell and HSL stimulus systems were not metrically identical, cross-experiment comparison was interpreted at the level of broad hue-family correspondence rather than as pointwise colorimetric equivalence. For the saturation manipulation, polynomial contrasts were used to test whether the rating pattern departed from monotonic increase and whether moderate conditions outperformed the highest-intensity treatment. Where pairwise comparisons were conducted, *p*-values were adjusted using the Benjamini–Hochberg false discovery rate procedure. Heatmaps, scanpaths, and other gaze-distribution figures were used descriptively alongside the condition-wise statistics rather than as stand-alone inferential tests.

Gender was examined only in supplementary analyses and is not treated as a primary explanatory factor in the present study. For the online replication and multi-viewpoint validation (Experiment 3), analyses were limited to directional consistency and rank-pattern stability, quantified using Spearman’s *ρ* between laboratory and online/viewpoint means, rather than strict inferential equivalence testing.

## 4 Results

### 4.1 Experiment 1: baseline of abstract color preference

**Figure 5:**
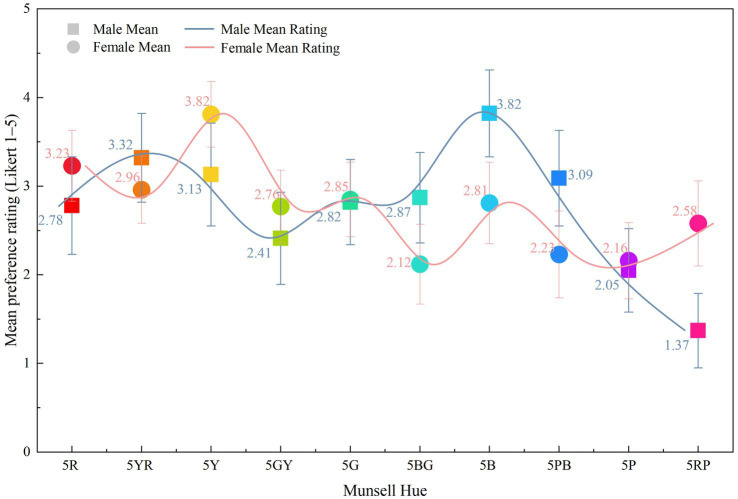
Mean esthetic preference scores for ten Munsell hues.

Experiment 1 established the decontextualized preference structure against which the later spatial evaluations were interpreted. When colors were presented as isolated Munsell samples rather than as environmental surfaces, participants showed a clear descriptive ordering of hue preference. As shown in Figure 5, yellow received the highest mean preference score (M = 4.32), followed by green-yellow and red, whereas red-purple received the lowest mean score (M = 2.15). Under these abstract viewing conditions, several warm or relatively vivid hues occupied the upper part of the preference distribution.

This baseline is important because it provides the reference pattern for evaluating contextual decoupling. In the absence of architectural embedding, participants responded to color as an isolated visual stimulus rather than as part of a workspace. The value of Figure 5 therefore lies less in the absolute ranking itself than in providing a decontextualized comparison point for the scene-based results reported later.

Descriptive gender-group differences were also visible at the abstract stage. Male participants tended to assign relatively higher ratings to parts of the blue spectrum, whereas female participants assigned relatively higher ratings to some warmer hues. These differences are reported only as descriptive tendencies, because the principal purpose of Experiment 1 was to establish a baseline ordering rather than to identify demographic mechanisms.

Overall, Figure 5 defines the pattern from which the rest of the study departs. The favorable standing of yellow and several other warm hues in the abstract task did not, by itself, justify their translation to large architectural surfaces. Instead, it set up the central empirical question of the study: whether a decontextualized preference structure would remain stable after color was embedded in a workspace context associated with sustained concentration and creative work.

### 4.2 Experiment 2: divergence of spatial contextual preference

**Figure 6:**
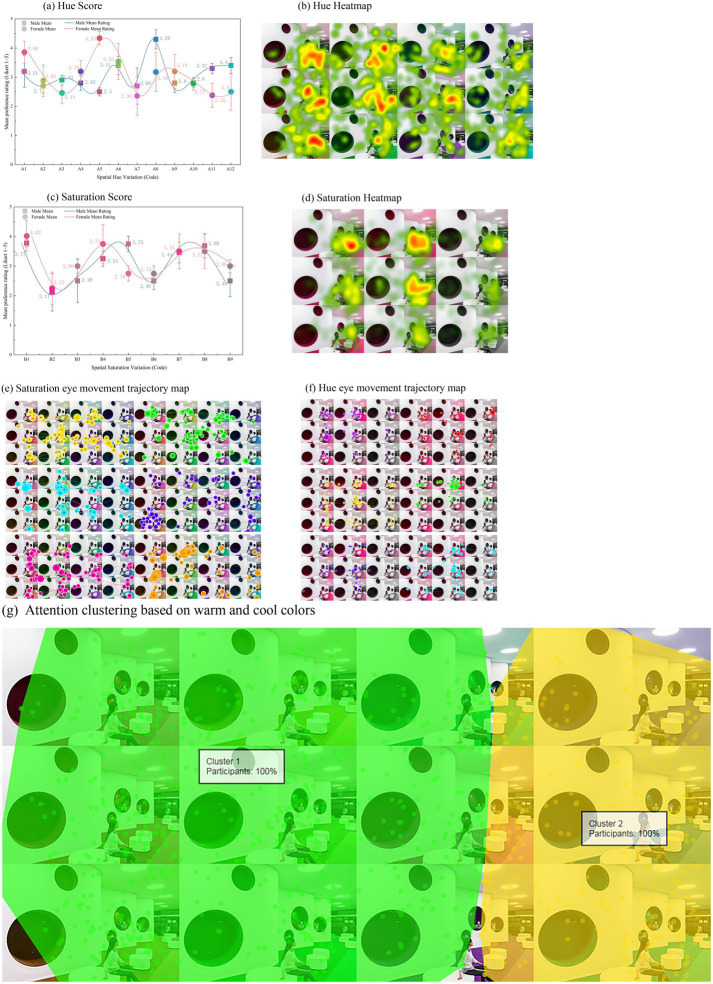
Multi-dimensional analysis of spatial color evaluation. **(a)** Mean spatial preference ratings for twelve hue conditions (A1-A12); error bars = SEM; connecting curve is a spline for visualization only. **(b)** Aggregated fixation-density heatmap across hue conditions; warmer regions indicate higher gaze density. **(c)** Mean spatial preference ratings for the nine saturation conditions (B1-B9); plotting conventions as in panel **(a)**. **(d)** Aggregated fixation-density heatmap across saturation conditions; color scale matched to panel **(b)**. **(e)** Representative scanpath for saturation conditions: circles denote fixations (diameter ∝ duration); lines denote saccades. **(f)** Representative scanpath for hue conditions; plotting conventions as in panel **(e)**. **(g)** Cluster-level fixation allocation grouped by warm vs. cool hue families; marker size reflects cluster mass.

A markedly different evaluative pattern emerged when color was judged as part of a simulated makerspace rather than as an isolated sample. As shown in Figure 6a, the ordering of spatial Preference across hue conditions no longer resembled the abstract pattern observed in Experiment 1. The principal result was therefore not simply a change in the highest-rated hue, but a broader contextual reordering of acceptability once color was evaluated as an environmental surface. This shift is also reflected in the descriptive statistics reported in Table 3. Within the hue set, A8 received the highest Preference and Comfort ratings (Preference: 3.74 ± 0.77, 95% CI [3.61, 3.87]; Comfort: 3.63 ± 0.77, 95% CI [3.50, 3.77]), whereas A7 showed the lowest Preference score (2.53 ± 0.67, 95% CI [2.41, 2.65]). A5 and A6 also remained in the more favorable range, while A2 and A3 remained in the lower part of the distribution. Taken together, Figure 6a and Table 3 indicate that the spatial hierarchy was reorganized rather than directly inherited from abstract color liking.

**Table 3:** Descriptive statistics by condition (Experiment 2).

Condition	Preference (M ± SD, 95% CI)	Comfort (M ± SD, 95% CI)	Fixation count (M ± SD)	Fixation duration (M ± SD)	Pupil change (M ± SD)
A1	3.53 ± 0.57, [3.43, 3.63]	3.52 ± 0.53, [3.43, 3.62]	14.0 ± 0.6	3913.5 ± 114.8	−5.18 ± 0.77
A2	2.79 ± 0.43, [2.72, 2.86]	2.72 ± 0.40, [2.65, 2.79]	14.8 ± 0.4	4144.6 ± 89.3	−5.75 ± 0.73
A3	2.68 ± 0.36, [2.62, 2.74]	2.69 ± 0.38, [2.62, 2.75]	17.0 ± 0.6	4751.1 ± 160.3	−7.07 ± 0.57
A4	3.00 ± 0.38, [2.93, 3.07]	3.01 ± 0.41, [2.94, 3.08]	10.2 ± 0.4	2849.4 ± 95.1	−2.62 ± 0.66
A5	3.42 ± 0.94, [3.26, 3.58]	3.33 ± 0.91, [3.17, 3.48]	13.4 ± 0.4	3749.7 ± 77.5	−3.76 ± 0.60
A6	3.47 ± 0.49, [3.38, 3.56]	3.35 ± 0.50, [3.26, 3.44]	11.4 ± 0.3	3180.8 ± 70.8	−4.19 ± 0.26
A7	2.53 ± 0.67, [2.41, 2.65]	2.60 ± 0.63, [2.49, 2.71]	13.8 ± 0.4	3856.1 ± 98.4	−3.60 ± 0.60
A8	3.74 ± 0.77, [3.61, 3.87]	3.63 ± 0.77, [3.50, 3.77]	8.0 ± 0.7	2249.6 ± 154.0	−0.77 ± 0.90
A9	3.00 ± 0.51, [2.91, 3.09]	3.03 ± 0.53, [2.94, 3.12]	17.2 ± 0.8	4803.2 ± 165.6	−6.82 ± 0.88
A10	2.79 ± 0.19, [2.76, 2.82]	2.84 ± 0.21, [2.81, 2.88]	11.6 ± 0.5	3242.0 ± 104.2	−3.75 ± 0.52
A11	2.84 ± 0.56, [2.74, 2.94]	2.75 ± 0.53, [2.66, 2.84]	10.4 ± 0.6	2912.6 ± 136.7	−3.21 ± 0.58
A12	2.95 ± 0.66, [2.83, 3.07]	2.87 ± 0.66, [2.76, 2.99]	12.3 ± 0.4	3455.6 ± 93.7	−4.91 ± 0.55
B1	3.90 ± 0.39, [3.83, 3.97]	3.81 ± 0.37, [3.75, 3.88]	13.4 ± 0.6	3758.2 ± 123.6	4.05 ± 0.29
B2	2.18 ± 0.59, [2.08, 2.28]	2.20 ± 0.60, [2.09, 2.30]	8.4 ± 0.6	2347.0 ± 150.2	−5.93 ± 0.31
B3	2.75 ± 0.60, [2.65, 2.85]	2.79 ± 0.59, [2.69, 2.89]	11.4 ± 0.3	3205.4 ± 60.1	1.37 ± 0.12
B4	3.50 ± 0.56, [3.40, 3.60]	3.46 ± 0.61, [3.36, 3.57]	11.0 ± 0.2	3091.5 ± 61.5	−1.18 ± 0.10
B5	3.25 ± 0.56, [3.15, 3.35]	3.22 ± 0.62, [3.11, 3.32]	9.8 ± 0.5	2743.8 ± 96.6	−3.39 ± 0.12
B6	2.62 ± 0.30, [2.57, 2.68]	2.58 ± 0.32, [2.53, 2.64]	10.4 ± 0.3	2898.8 ± 57.4	−2.05 ± 0.21
B7	3.48 ± 0.48, [3.40, 3.56]	3.45 ± 0.46, [3.37, 3.53]	12.2 ± 0.3	3417.6 ± 59.6	0.68 ± 0.15
B8	3.59 ± 0.51, [3.51, 3.68]	3.48 ± 0.56, [3.38, 3.57]	13.0 ± 0.3	3649.6 ± 59.3	−0.58 ± 0.18
B9	2.75 ± 0.48, [2.67, 2.83]	2.71 ± 0.43, [2.63, 2.78]	8.8 ± 0.3	2475.7 ± 68.5	−2.67 ± 0.10

This reordering was especially visible for the yellow-related hue family. In Experiment 1, yellow belonged to the more favorably evaluated abstract hues, whereas under scene-based evaluation the corresponding broad hue family no longer occupied the upper end of the spatial Preference pattern. Consistent with the broad hue-family interpretation adopted in Section 3, this indicates a family-level reorganization rather than a pointwise colorimetric reversal.

The gaze-distribution results further indicate that spatial evaluation was not reducible to visual saliency alone. As illustrated in Figure 6b, conditions associated with dense visual attention were not necessarily those that received the highest Preference or Comfort ratings. This dissociation becomes clearer when the heatmap pattern in Figure 6b is considered together with the rating profile in Figure 6a and the corresponding values in Table 3. For example, A3 and A9 attracted the highest levels of gaze allocation, with fixation counts of 17.0 ± 0.6 and 17.2 ± 0.8 and fixation durations of 4751.1 ± 160.3 ms and 4803.2 ± 165.6 ms, respectively, yet neither condition ranked highly in subjective evaluation. By contrast, A8, which received the strongest Preference and Comfort ratings, showed the lowest fixation count (8.0 ± 0.7) and one of the shortest fixation durations (2249.6 ± 154.0 ms). These comparisons indicate that attention capture and positive spatial appraisal did not move together uniformly across hue conditions.

The saturation manipulation yielded a similarly informative pattern. Figure 6c shows that the most intense condition was evaluated less favorably than several moderate saturation conditions. The descriptive statistics in Table 3 support this non-monotonic result. B1 received the highest ratings (Preference: 3.90 ± 0.39, 95% CI [3.83, 3.97]; Comfort: 3.81 ± 0.37, 95% CI [3.75, 3.88]), whereas B2 showed the lowest ratings (Preference: 2.18 ± 0.59, 95% CI [2.08, 2.28]; Comfort: 2.20 ± 0.60, 95% CI [2.09, 2.30]). B4, B7, and B8 also remained more favorable than the lower-rated extremes. Spatial evaluation therefore did not improve monotonically with chromatic intensity; instead, several moderate conditions were judged more positively than the most intense treatment.

A related dissociation was visible in the eye-tracking summaries for the saturation set. As shown in Figure 6d, visually intense conditions could still attract substantial attention without being judged as especially supportive of sustained work. The scanpath visualizations in Figures 6e–g provide a descriptive complement to the rating and heatmap panels by illustrating differences in gaze organization and attentional clustering across hue and saturation regimes. These panels are not interpreted as stand-alone inferential tests. At the pattern level, warmer or more intense conditions often produced denser visual exploration, whereas cooler or more moderate conditions more often aligned with favorable environmental appraisal. Taken together, Figure 6 and Table 3 indicate that once color was embedded in a makerspace-like context, the original abstract preference structure was reorganized rather than preserved.

### 4.3 Physiological patterns relevant to the multimodal interpretation

The eye-tracking results showed that visual attention and positive spatial evaluation did not move together uniformly across conditions. Pupillometric data were therefore examined to determine whether this dissociation was accompanied by a condition-sensitive physiological pattern under luminance-controlled viewing. Because pupil size is influenced by multiple processes, baseline-normalized pupil change was not interpreted as a direct readout of cognitive load. Instead, it was treated as one physiological component within a broader multimodal response profile.

**Figure 7:**
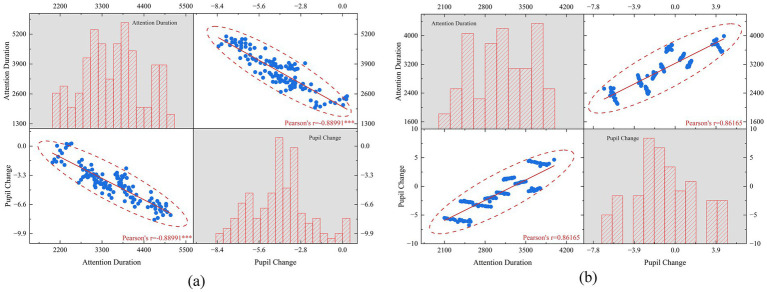
Visual-pupillary pattern summaries across chromatic regimes: **(a)** higher-intensity warm-hue or hypersaturation conditions showing stronger attention capture without correspondingly favorable evaluation; **(b)** cooler-hue or moderate-saturation conditions showing a comparatively more aligned multimodal profile.

As illustrated in Figure 7, the relation between fixation duration and pupil response differed across chromatic regimes. Figure 7a summarizes the high-intensity warm-hue and hypersaturation regime, in which stronger visual engagement did not coincide with the more favorable evaluative profile observed in the rating results. This interpretation is consistent with the descriptive statistics in Table 3: conditions such as A3 and A9 showed long fixation durations (4751.1 ± 160.3 ms and 4803.2 ± 165.6 ms) together with strongly negative pupil-change values (−7.07 ± 0.57 and −6.82 ± 0.88), yet both remained modest or low in Preference and Comfort. By contrast, A8 combined the most favorable subjective evaluation with the lowest fixation count (8.0 ± 0.7), one of the shortest fixation durations (2249.6 ± 154.0 ms), and the least negative pupil-change value (−0.77 ± 0.90). Across these conditions, attention allocation, subjective evaluation, and pupil response were not uniformly aligned.

Figure 7b summarizes a cluster of cooler-hue or moderate-saturation conditions for which the relation among attention, self-report, and pupil response appeared comparatively less discordant. This reading is consistent with the more favorable rating pattern observed for moderate saturation conditions such as B1, B4, B7, and B8 in Table 3. In these conditions, subjective acceptance was comparatively stronger and the multimodal pattern was less evidently opposed than in the higher-intensity regime. The contrast between Figures 7a,b therefore indicates that the coupling among looking behavior, evaluative judgment, and physiological response changed across conditions rather than remaining uniform throughout the stimulus set.

The omnibus analyses summarized in Table 2 show that these visual and physiological differences were not anecdotal. Hue significantly affected Preference, Comfort, fixation count, fixation duration, and pupil change, and saturation also showed significant effects across all five outcomes. Figure 7 provides a pattern-level visualization of these differences, whereas Table 2 establishes that the condition effects were statistically robust at the omnibus level.

Taken together, Figure 7 and Table 2 support a cautious multimodal interpretation of the findings. One aspect of the pattern concerns attentional saliency, namely whether a chromatic condition attracts gaze because it is visually prominent within the scene. Another aspect concerns context-sensitive appraisal, namely whether the same chromatic atmosphere is judged as compatible with concentration, comfort, and sustained use. The present results do not isolate a single underlying mechanism. More narrowly, they indicate that chromatic conditions differentiated not only what participants preferred and looked at, but also the physiological profile observed under controlled viewing conditions.

### 4.4 Spatial robustness across viewing perspectives

**Figure 8:**
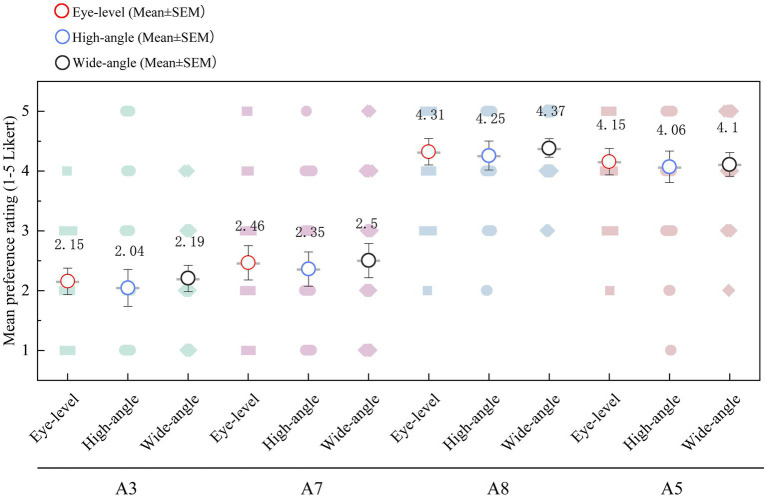
Validation of preference stability across different spatial viewpoints.

The final validation stage examined whether the observed preference structure depended primarily on a single camera framing. Figure 8 addresses this issue by presenting four target hue conditions across three viewpoints: eye-level, high-angle, and wide-angle. The purpose of this analysis was not to reproduce the full laboratory protocol, but to test whether the broad ranking pattern identified earlier remained visible when the same color-treated environments were viewed from different spatial perspectives.

Across viewpoints, the more favorably evaluated conditions, especially A8 and A5, remained near the upper end of the rating range, whereas the lower-rated comparison conditions, A3 and A7, remained substantially less preferred. This pattern indicates a modest but relevant degree of robustness. The main spatial preference contrast remained visible after the scene was re-rendered from different perspectives, which reduces the likelihood that the earlier findings were driven solely by one particular compositional arrangement in the original image.

At the same time, Figure 8 should not be used to claim strict viewpoint invariance, because no formal hue-by-viewpoint interaction is inferred here. Contextual decoupling was not confined to a single camera angle, and the broad spatial preference pattern remained recognizable under alternative viewpoints, providing supportive but not conclusive evidence of robustness.

## 5 Discussion

### 5.1 H1: contextual reordering between abstract and spatial preference

H1 proposed that preference orderings observed under abstract viewing conditions would not remain stable when comparable chromatic conditions were evaluated in a makerspace-like environment. The present results are consistent with this expectation. The key finding was not merely that participants selected different colors across two tasks, but that the evaluative structure changed once color was judged as part of an enclosing workspace rather than as an isolated sample.

The observed contextual reordering sits within a small but growing literature that has already pointed to something similar without labeling it. Ertez Ural and Yilmazer (2010) showed that when the same colors were evaluated through sequential media — chips, two-dimensional renderings, and three-dimensional spatial simulations — the semantic profile of each color changed systematically with the medium of presentation, implying that the task of ‘evaluating color’ is not invariant across presentation contexts. Akbay and Demirbaş (2023) framed an even closer distinction, contrasting context-free and in-context spatial color preferences and reporting that the two differ both in level and in how they relate to individual-difference constructs such as extraversion. The present study extends these findings in two ways. First, it adds multimodal evidence: the divergence between abstract and spatial preference is not only visible in self-report but is also accompanied by systematic differences in attentional distribution and baseline-normalized pupil response. Second, it situates the divergence in a cognitively demanding educational setting, where color must also carry a functional signal of suitability for sustained work. Contextual decoupling is best understood as a refinement of existing phenomena, clarifying where abstract preference fails to transfer and how that failure can be detected across convergent indicators.

Abstract preference measures retain value, but they should not be transferred uncritically into environmental design recommendations. Because the cross-experiment comparison is interpreted at the level of broad hue-family correspondence rather than pointwise colorimetric equivalence, the present evidence supports contextual reordering rather than exact color-by-color reversal.

### 5.2 H2: nonlinear effect of saturation on spatial evaluation

H2 proposed that spatial Preference and Comfort would vary nonlinearly with saturation, with one or more moderate conditions outperforming the most intense treatment. The present results are consistent with this prediction. Preference and Comfort did not increase monotonically with chromatic intensity. Instead, the highest-intensity treatment showed a clear evaluative decline relative to several moderate saturation conditions.

This is more informative than a simple statement that hypersaturation was disliked. It indicates that saturation in a workspace-like setting functions as a bounded design variable rather than as a straightforward more-is-better dimension. Within the present stimulus set, increasing intensity did not continuously improve appreciation or comfort. Beyond a certain range, it appeared to reduce environmental acceptability.

The study does not establish a universal saturation threshold nor does it justify a precise prescription for all makerspaces. In the present context, the relation between saturation and spatial evaluation was nonlinear, and the most intense treatment was not the optimal one.

### 5.3 H3: dissociation between attentional saliency and positive appraisal

H3 proposed that visually intense conditions would attract attention without necessarily receiving more favorable spatial evaluations. The present findings are consistent with this expectation. Across both hue and saturation manipulations, some of the most visually prominent conditions drew substantial gaze allocation while failing to produce correspondingly high Preference or Comfort ratings.

This pattern separates two constructs that are often conflated in applied design reasoning. A color can be visually dominant, memorable, or difficult to ignore and still be judged as excessive, tiring, or poorly matched to the intended use of the space. Saliency is not equivalent to environmental success.

The findings therefore reinforce the broader argument of the paper. Contextual decoupling is not simply random inconsistency in taste. More narrowly, it reflects a recurring mismatch between what captures attention and what supports positive spatial judgment under the present task conditions.

### 5.4 H4: condition-sensitive physiological differences under luminance control

H4 proposed that, under luminance-controlled conditions, baseline-normalized pupil dynamics would vary across chromatic conditions and provide convergent physiological evidence relevant to the interpretation of spatial evaluation. The present results are consistent with this expectation, but the support is necessarily cautious.

The important point is convergence rather than over-interpretation. The pupillometric patterns did not stand alone; their value lies in the fact that they varied with chromatic regime and could be interpreted alongside the gaze and rating results. In the more visually intense conditions, the multimodal pattern was less compatible with favorable spatial evaluation. In the cooler or more moderate conditions, attention, self-report, and pupil response appeared comparatively less discordant.

Pupil response cannot be treated as a pure measure of cognitive load, because pupil dynamics are influenced by multiple factors, including luminance history, arousal, uncertainty, task demand, and preprocessing decisions. The appropriate conclusion is therefore limited: the physiological data were condition-sensitive and compatible with a visual-processing-demand-related interpretation, but they do not by themselves isolate a unique latent mechanism.

### 5.5 H5: gender-group differences as a supplementary observation

H5 concerned the expectation that gender-group differences visible in abstract color preference would appear less pronounced when color was evaluated in spatial context. The present data are descriptively consistent with this expectation, but they do not warrant a formal confirmatory claim in the absence of full interaction statistics.

The observed pattern suggests that once participants judged color as part of a makerspace-like environment, broader contextual considerations may have become more influential than the group differences that were more visible in the abstract task. This interpretation is theoretically plausible because spatial evaluation can shift judgment away from isolated liking and toward suitability, comfort, and task compatibility.

The current evidence does not support the stronger claim that gender differences disappear or become irrelevant. Such differences appeared less decisive under spatial evaluation than under decontextualized color judgment and are treated here as exploratory.

### 5.6 Design implications and practical contributions

Our stimuli were static, luminance-controlled simulations of makerspace-like scenes viewed for 8 s; color was applied broadly to major interior surfaces rather than in spatially differentiated roles; and participants were drawn mainly from one cultural and disciplinary population. Consequently, the present data do not license general claims about color in makerspaces as a class, about long-duration occupancy, or about applications in which color is deployed as a localized accent or wayfinding element.

Within these boundaries, the results point to a restrained observation: under short-duration evaluation of simulated makerspace-like scenes in which chromatic treatments occupied major surfaces, cooler and more moderate chromatic conditions were more consistently associated with favorable Preference and Comfort ratings than highly intense warm or hypersaturated conditions. This pattern is tied to the paradigm employed and is not advanced as a universal palette prescription; its generalization to longer occupancy, to immersive or built settings, and to broader populations remains to be established.

Because color was never manipulated as a localized element, our data cannot adjudicate whether warm or hypersaturated hues would function differently when used as accents or wayfinding markers rather than as background fields. The co-occurrence of strong attentional capture with modest subjective evaluation under high-intensity conditions is compatible with — but does not demonstrate — such a role-dependent effect; we accordingly treat it as a hypothesis for subsequent studies in which the spatial role of color is explicitly varied.

At the procedural level, the dissociation between swatch-based and spatially contextualized preference observed here reinforces the case, already implicit in previous work (Ertez Ural and Yilmazer, 2010; Akbay and Demirbaş, 2023), for evaluating consequential interior palettes in spatially embedded representations rather than extrapolating from isolated samples.

## 6 Conclusion

This study examined whether color preference established under abstract viewing remained stable when color was evaluated as part of a makerspace-like interior, and combined self-report, eye-tracking, and pupillometric evidence to probe that question. Across the three experiments, the preference structure observed for isolated color samples did not transfer directly to the spatial task: colors rated favorably in abstract form were not necessarily preferred on large architectural surfaces, whereas cooler or moderate chromatic conditions were more consistently evaluated as compatible with sustained cognitive work. Attention capture and positive spatial appraisal dissociated across conditions, and pupillometric differences were condition-sensitive in a manner compatible with variation in visual processing demand. The findings support contextual decoupling as a refinement of abstract color-preference research, and show that multimodal convergence can provide useful discipline on the interpretation of environmental color.

The conclusions above are bounded by four delimitations. (i) Exposure duration. The stimuli were static simulated scenes viewed for 8 s, and generalization from short-duration exposure to long-duration occupancy remains to be tested. (ii) Sample composition. Participants were drawn mainly from Chinese university populations in design and engineering-related programs; cultural, age-group, and disciplinary generalizability should therefore be examined before broader claims are made, particularly in light of evidence that color–emotion associations are shaped by both universal and culture-specific components (Jonauskaite et al., 2020; Hurlbert and Ling, 2007). (iii) Display medium. Multimodal convergence was obtained under luminance-controlled monitor viewing rather than in immersive VR or real built environments; pupillometric effects in particular are sensitive to background luminance (Pan et al., 2024) and should be re-examined in more naturalistic settings before stronger mechanistic claims are made. (iv) Colorimetric correspondence. Cross-experiment comparison was interpreted at the level of broad hue-family correspondence rather than pointwise colorimetric equivalence because Munsell-based and HSL-based stimuli are not metrically identical. Future work should extend the design to longer exposure, navigable virtual environments, and real built settings, and should combine the present multimodal approach with individual-difference constructs to clarify how personality and context interact in shaping spatial color response.

## Data Availability

The datasets presented in this study can be found in online repositories. The names of the repository/repositories and accession number(s) can be found in the article/Supplementary material.
