# Supplementary material for: Contextual decoupling in color preference: multimodal evidence from spatial evaluation in makerspaces
Source: Front Psychol. 2026 May 15;17:1826920. doi: 10.3389/fpsyg.2026.1826920 (PMC13218920; doi:10.3389/fpsyg.2026.1826920)
Supplement: Supplementary file 1 [file Table_1.DOCX]

**Table S1: Experiment 1 planned pairwise contrasts (Abstract Preference)**

| Contrast | Mean Difference | SE | df | t-ratio | p-value (FDR adjusted) | 95% CI |
| --- | --- | --- | --- | --- | --- | --- |
| Yellow (5Y) - Red-Purple (5RP) | 2.17 | 0.14 | 1125 | 15.50 | < .001 | [1.89, 2.45] |
| Yellow (5Y) - Purple (5P) | 1.85 | 0.14 | 1125 | 13.21 | < .001 | [1.57, 2.13] |
| Yellow (5Y) - Purple-Blue (5PB) | 1.68 | 0.14 | 1125 | 12.00 | < .001 | [1.40, 1.96] |
| Green-Yellow (5GY) - Red-Purple (5RP) | 1.45 | 0.14 | 1125 | 10.35 | < .001 | [1.17, 1.73] |
| Red (5R) - Red-Purple (5RP) | 1.12 | 0.14 | 1125 | 8.00 | < .001 | [0.84, 1.40] |

**Table S2: Experiment 2 selected hue contrasts (Spatial Preference)**

| Contrast | Mean Difference | SE | df | t-ratio | p-value (FDR adjusted) | 95% CI |
| --- | --- | --- | --- | --- | --- | --- |
| A8 (Cooler/Blue) - A3 (Warm/Yellow) | 1.06 | 0.10 | 1500 | 10.60 | < .001 | [0.86, 1.26] |
| A8 (Cooler/Blue) - A7 (Red-Purple) | 1.21 | 0.10 | 1500 | 12.10 | < .001 | [1.01, 1.41] |
| A5 (Cooler/Red) - A3 (Warm/Yellow) | 0.74 | 0.10 | 1500 | 7.40 | < .001 | [0.54, 0.94] |
| A5 (Cooler/Red) - A7 (Red-Purple) | 0.89 | 0.10 | 1500 | 8.90 | < .001 | [0.69, 1.09] |

**Table S3: Experiment 2 saturation contrasts (Spatial Preference & Comfort)**

| Metric | Contrast | Mean Diff. | SE | df | t-ratio | p-value (FDR) | 95% CI |
| --- | --- | --- | --- | --- | --- | --- | --- |
| Preference | B1 (Baseline) - B2 (Hypersaturated) | 1.72 | 0.08 | 1125 | 21.50 | < .001 | [1.56, 1.88] |
| Preference | B4 (Moderate) - B2 (Hypersaturated) | 1.32 | 0.08 | 1125 | 16.50 | < .001 | [1.16, 1.48] |
| Preference | B1 (Baseline) - B9 (Achromatic) | 1.15 | 0.08 | 1125 | 14.37 | < .001 | [0.99, 1.31] |
| Comfort | B1 (Baseline) - B2 (Hypersaturated) | 1.61 | 0.09 | 1125 | 17.88 | < .001 | [1.43, 1.79] |
| Comfort | B4 (Moderate) - B2 (Hypersaturated) | 1.26 | 0.09 | 1125 | 14.00 | < .001 | [1.08, 1.44] |

**Table S4: Experiment 3 replication statistics (Spatial Preference)**

| Hue Condition | Independent Sample Mean (N=112) | SD | Correlation (Pearson's r) with Exp 2 Means | p-value |
| --- | --- | --- | --- | --- |
| A8 | 3.65 | 0.81 | 0.85 (across all 12 hues) | < .001 |
| A5 | 3.31 | 0.85 | - | - |
| A3 | 2.55 | 0.72 | - | - |
| A7 | 2.48 | 0.76 | - | - |
